# Fabrication of Polymer Composite Fibers Embedding Ultra-Long Micro/Nanowires

**DOI:** 10.3390/nano11040939

**Published:** 2021-04-07

**Authors:** Bo Yang, Dawei Pan, Laixi Sun, Shufan Chen, Weidong Wu, Bo Li

**Affiliations:** 1 Research Center of Laser Fusion, China Academy of Engineering Physics, Mianyang 621900, China; fayeyang00@163.com (B.Y.); 13668244686@163.com (D.P.); sunlaixi@126.com (L.S.); wuweidonging@163.com (W.W.); lib6711@163.com (B.L.); 2 IFSA Collaborative Innovation Center, Shanghai Jiao Tong University, Shanghai 200240, China

**Keywords:** polymer composite fibers, micro/nano porous array templates, ultra-long micro/nanowires, melt co-drawing, Cu nanowires

## Abstract

Fabrication of polymer composite fibers embedding ultra-long micro/nanowires via an iterative melt co-drawing and bundling technique is reported in this study. The poly(methyl methacrylate) (PMMA) porous array templates were prepared with section-cutting the PMMA/polystyrene (PS) (shell/core) composite fibers and dissolution of inner PS. The results showed that the PS cores or pores in the PMMA matrix are regularly arranged with hexagonal, and their diameter and spacing exhibits a uniform distribution. Especially, the core diameter can be precisely controlled from millimeter-scale to nanometer-scale by multi-step melt co-drawing. Based on the PMMA porous array templates, the Cu nanowires were successfully prepared by electrochemical deposition. Moreover, to fabricate PMMA ultra-long micro/nanowires, the composite fibers with converse shell/core component of PS/PMMA were initially prepared, and then the outer PS was dissolved. The obtained PMMA micro/nanowires were characterized with smooth complete orientation structure. The study provides an experimental basis for fabricating such polymer composite fibers, micro/nano porous array templates, and micro/nanowires with precise and controllable manner to meet the real application requirements.

## 1. Introduction

Micro/nanowires have been attracting a great deal of attention for decades because they are arguably the most studied micro/nano material model to make functional devices [1,2,3,4]. For example, micro/nano porous array polymer template is always used to prepare the metallic micro/nanowires, which plays a significant role in the regulation of structure and size parameters of metal micro/nanowires [5,6]. The micro/nanowires or composite fibers embedded with micro/nanowires are attractive building blocks for fabricating functional devices, especially complex device arrays. Ultra-long micro/nanowires are also used to design and integrate large-area, flexible, and high-density device components, such as high-performance semiconductors on flexible polymers [7], implantable or wearable chemical and biological sensing [8], large-area photodetectors, optical phased array, and micro/nano fluidics channels, because they could benefit overall integration by facilitating interconnection of nano-electronic device arrays to large scale input/output wires in a system [9]. Note that, among the involved applications, the size and morphology of micro/nano structures have a significant impact on the characteristics of the resulting materials or functional devices [10,11,12]. 

At present, two kinds of methods, regarding template method and non-template method, have been developed to prepare such micro/nano structures. Generally, the template method is widely employed to fabricate micro/nano materials with special morphologies, such as nanowires and nanotubes. By using template as a carrier, the size, shape, structure, and properties of micro/nano materials can be accurately controlled. This relatively simple preparation process is very suitable for mass production. However, with the increasing demand for high-precision performance of micro/nano functional devices, one technically challenging preparation of the desired micro/nano structures with specific micro/nano structure parameters (e.g., diameter, spacing, length, and their distribution) is to enhance the designability and controllability. It is therefore essential to achieve the precise control over them by putting forward an optimal and most cost-effective method.

During the preparation process of micro/nanowires using template method, one of the most important issues is to obtain porous template with a specific geometric parameter at a wide range. For this purpose, porous anodic aluminum oxide (AAO) template where its pores can reach tens to hundreds of nanometers is always employed to fabricate the ordered nanowires [13,14,15]. Recently, AAO templates, produced by one step aluminum anodization and without the sputtering of a conductive metals on one side of the membrane, were used to produce metallic nanowires up to 20 μm allowing for its application in several electrochemical devices [16,17]. However, it should be noted that, this method fails to form a micron-scale array pore structure because of the inherent characteristics of its self-organization mechanism [18,19,20,21]. To obtain porous templates with diameter larger than several dozen micrometers, laser drilling is proposed. Due to the uneven energy distribution of laser, the shape of the obtained hole by such method is often irregular [22,23,24,25]. Moreover, other methods for preparing the porous template are also investigated. Heavy-ion track etching technology can be used to prepare polymer templates with nanoporous structure. It is a pity that the obtained pores randomly distribute and their diameter or spacing can only be presented as a statistic result [26]. Although photolithographic techniques can provide great controllability over the position and orientation of structures, the length–diameter ratio of nanowires is strongly constrained by the span–depth ratio of the photoetching [27]. In a word, among the existing preparation methods, it is difficult to obtain porous array template at a cross-scale range from millimeter-scale to nanometer-scale, and further the geometric parameters of the resulting porous array template cannot be designed with a controllable and precise manner.

To achieve the preparation of ultra-long nanowires, a great number of methods are proposed. The electrospinning is usually used to prepare such ultra-long nanowires, but it is difficult to produce ordered or oriented micro/nanowires [28,29]. Many direct-write techniques and related improvements have also been developed to fabricate a variety of nanowires and nanofibers, but there still lacks the capability to fabricate large scale controllable micro/nanowires [30]. For the chemical synthesis method, the required operation temperature is always high, and the post-conversion process is complex, leading to a difficulty in the preparation of ultra-long nanowires [31]. Another well-established method is reported for fabricating ordered long nanowire or nanotube arrays in a flexible polymer fiber [32]. The size parameters of the resulting nanowires can be effectively controlled by using such method, but it is confined to the fabrication of aligned long multilateral multifunctional fibers [33]. Although many fabrication methods of ultra-long nanowires have been proposed, few studies focus on controlling the size parameters of the micro/nano structures. Especially, it is also very difficult to obtain the micro/nano structures with multi-scale size, always resulting in a limitation in real applications.

For these reasons mentioned above, a simple method, namely multi-step melt co-drawing techniques [5,6,34], is proposed to prepare the micro/nano porous array polymer templates and ultra-long polymer micro/nanowires. Compared with other conventional methods, this technique possesses many advantages: (1) the size parameters of micro/nano structures can be easily controlled in a large range (from millimeter-scale to nanometer-scale); (2) the obtained arrangement morphology of the micro/nano structures exhibits excellent uniformity; (3) high yield, high repeatability, easy operation; and (4) no being limited to polymer materials. Furthermore, by selecting appropriate melt viscosity and thermal properties, such multi-step melt co-drawing technique can also be applied to manufacture other metals or alloys.

In this study, a suitable core/shell material system of the preform was initially selected according to their thermal, rheological, and solubility properties, and then the PMMA/PS preform was melt co-drawn to form polymer fiber. After the cross-section and dissolution of inner PS, the PMMA micro/nano porous array templates can be fabricated. By adjusting the co-drawing steps and experimental parameters, the geometric sizes of pores in the PMMA template can be precisely controlled. Based on the PMMA templates, an electrochemical deposition technique was used to prepare the Cu nanowires. Instead, when the PS and PMMA were selected as the shell and core materials, a similar preparation process can be used to prepare the PMMA ultra-long micro/nanowires. Moreover, the surface morphology of the resulting PMMA micro/nano porous array templates and PMMA ultra-long micro/nanowires was also characterized.

## 2. Materials and Methods

### 2.1. Materials

In this study, commercial PS and PMMA rods were used to prepare the preformed rod. PS1 (M_w_ ~ 310,000 g/mol) and PS2 (M_w_ ~ 240,000 g/mol) rods were purchased from Shanghai Yanqiao engineering plastics. Co., Ltd (Shanghai, China). PMMA1 (M_w_ ~ 120,000 g/mol) and PMMA2 (M_w_ ~ 300,000 g/mol) rods were purchased from Taiwan Qimei Co., Ltd. (Tainan, Taiwan). Cyclohexane (purity ≥ 99.5 wt%) and tetrahydrofuran (purity ≥ 99.0 wt%) solutions, used for the dissolution of PS and PMMA, were obtained from Guangdong Guanghua Sci-Tech Co., Ltd. (Shantou, Guangdong, China) and Tianjing Kermel chemical reagent Co. Ltd. (Tianjin, Hebei, China), respectively.

### 2.2. Sample Preparation

**Figure 1:**
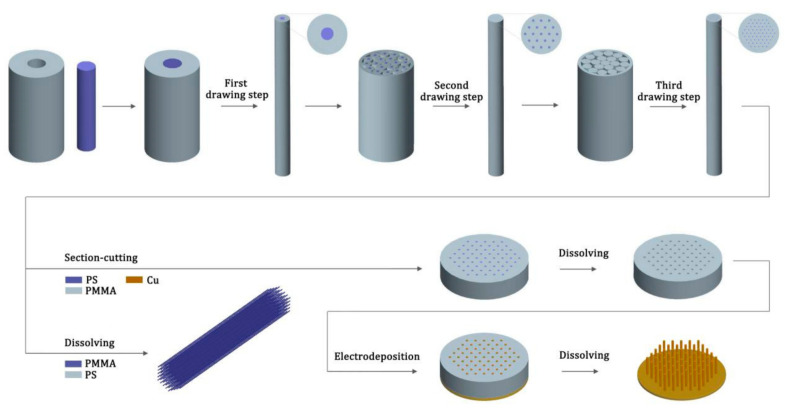
Schematic diagram of the fabrication procedure for the poly(methyl methacrylate) (PMMA) porous array templates, Cu micro/nanowires and ultra-long PMMA micro/nanowires.

The schematic illustrations of the fabrication process for PMMA porous array templates, Cu micro/nanowires, and ultra-long PMMA micro/nanowires are shown in Figure 1. As for the PMMA porous array template and Cu micro/nanowires preparation, the PS rod (colored in blue) was inserted into PMMA tube (colored in grey) to make the preformed rod for the first drawing step. Note that, the inner diameter of PMMA tube should match with the outer diameter of PS rod. After the first drawing, the obtained PMMA/PS fibers were cut into short pieces with equal length, which were stacked together to form a hexagonal bundle in a thin-wall PMMA tube for the next drawing. By repeating the drawing-cutting-stacking process for several times, the PMMA composite fibers embedded with ordered arrays of PS micro/nanowires were obtained.

After that, the composite fiber was cut perpendicular to its axis, forming the slices with a specific required thickness. To form a PMMA porous array template with a specific thickness, the fiber was initially cut into slices with a thickness of several hundred microns using a diamond wire cutting machine, and then the slice underwent double-faced grinding and polishing to form a thin slice with a thickness of tens of micrometer, finally the PS was dissolved in cyclohexane solution at approximately 60 °C.

Based on the PMMA porous array templates, Cu or other metallic micro/nanowires can be prepared by the electrochemical deposition technique. Generally, the electrochemical deposition process mainly consists of three main steps. First, an ultrapure Cu film with a thickness of approximately 4 µm was deposited on one side of the template via magnetron sputtering at a working pressure of 1.0 × 10^−4^ Pa and input power of 40 W for approximately 8 h. Then, the conducting wire was connected to the metallic film to serve as an electrode. To prevent margin deposition of the template, we covered the surrounding part of the template with insulating tape so that only the porous part was exposed. Finally, the sample was dipped into the electrolyte for the deposition processing. A 500 mL deionized water solution of electrolyte was prepared with 100 g CuSO_4_, 30 g H_2_SO_4_, 49.5 mg NaCl, 0.2 mL New Kotic-A, and 2 mL New Kotic-C. The electrochemical deposition was carried out at 0.3 V vs. saturated calomel electrode (SCE) and a 1 Hz direct current pulse for 100–200 s. After the deposition process, the Cu or other metallic micro/nanowires can be obtained through dissolving the PMMA template in the tetrahydrofuran solution. 

As for the preparation of PMMA micro/nanowires, it can be achieved by simply changing the position of the two polymer materials. The PMMA rod (colored in blue) was inserted into PS tube (colored in grey) to make the preformed rod. The main step regarding drawing maintain unchanged. The obtained PS/PMMA composite fibers were soaked in a cyclohexane solution to dissolve the PS at approximately 60 °C. The extracted PMMA micro/nanowires samples were further rinsed gently with cyclohexane to remove residual PS.

During the experiment, a special drawing system was used to carry out the fiber drawing procedures. The system mainly consists of feeding, heating furnace, die, laser micrometer, and capstan units. Firstly, the preformed rod was placed into the heating furnace and preheated at a proper temperature. Then, the melted preform was extruded from the die with a certain size into a fine flow while feeding the preform downward at a certain feeding speed. The flow moved downward through the laser micrometer, and was finally collected by a capstan with a certain drawing speed under a tensile force applied by the tensile motor. Smaller fiber outer diameters could be obtained with a relatively smaller die size (0.5 mm), lower feeding speed (0.25–0.5 mm/min), and higher drawing speed (900–1800 mm/min). Larger fiber outer diameters could be obtained with a relatively larger die size (1–3 mm), higher feeding speed (1–3 mm/min), and lower drawing speed (300–900 mm/min).

### 2.3. Characterisation

The morphologies of the PMMA template with micrometer and nanometer porous arrays and the corresponding PMMA micro/nanowire were characterized by optical microscope and scanning electron microscopy (SEM). Note that, during the SEM observation process, the surface of the obtained materials must be coated with Au to achieve electric conduction. Surface elements were analyzed using energy dispersive X-ray spectroscopy (EDX) with an acceleration voltage of 15 kV. To obtain the optimal matching relationship between the PS and PMMA materials, a differential scanning calorimeter (DSC) was utilized to measure the glass transition temperatures of the samples. The samples were heated from 30 to 250 °C at a rate of 10 °C/min in a nitrogen atmosphere, and then kept at 250 °C for 5 min to eliminate the thermal history. After that, the samples were cooled to 30 °C at a rate of 10 °C/min, held at 30 °C for 5 min, and then heated to 250 °C at a heating rate of 10 °C/min. The constant nitrogen flow rate was 50 cm^3^/min. Moreover, the shear viscosity of the materials was tested by a capillary rheometer at 220, 230, and 240 °C, respectively.

## 3. Results and Discussion

To successfully prepare the micro/nano porous array templates and micro/nanowires with various components, the selecting and matching of the preformed core and shell materials systems is one of the most important issues to be addressed. During the co-extrusion moulding process, the flow stability of the core and shell materials has a significant impact on the interface of array structure, and it requires a close shear viscosity under the appropriate temperature and shear rate. As a result, the thermomechanical properties, regarding thermal deformation temperature and rheological properties of core and shell materials should be initially well matched. The materials of the core and shell, on the other hand, should be selectively dissolved. In detail, for the preparation of micro/nano porous array template, the core material needs to be removed such that polymer porous structure can be obtained. Of course, the dissolution process of core material is required to have an ignorable impact on the shell material property. Moreover, to fabricate the polymer micro/nanowires, the solubility of core and shell materials just exhibits an opposite rule. Note that, the dissolution issue is not required during the fabrication of composite fiber embedded with micro/nanowires. 

**Figure 2:**
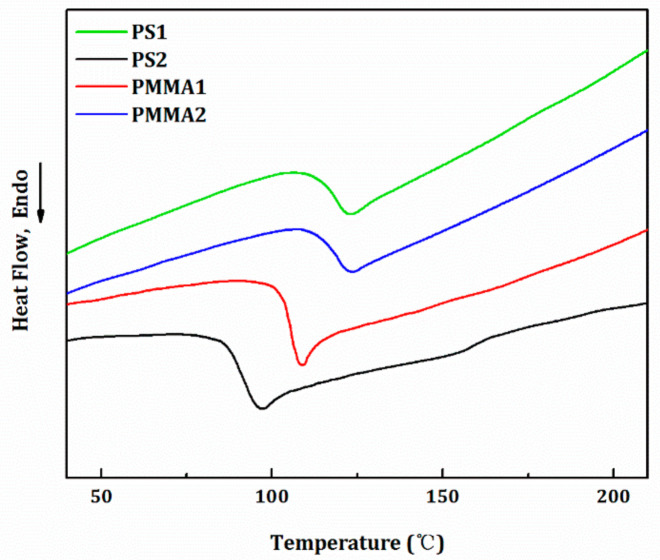
The differential scanning calorimetry heating curves of the PS1, PS2, PMMA1 and PMMA2 materials.

The two kinds of PMMA (named PMMA1 and PMMA2) and PS (named PS1 and PS2) are employed in this study as the alternative raw materials. To select a suitable material system, the thermal and rheological properties of these materials were tested and analyzed. The DSC testing results are shown in Figure 2, and the results show that, the glass transition temperatures of PMMA1, PMMA2, PS1, and PS2 were 102, 109, 108, and 85 °C, respectively. It can be found that glass transition temperatures of PMMA2 and PS1 were close, indicating an existence of optimal matching relationship between them. As a result, the PMMA2 and PS1 can be used as the preformed rods for the melt co-drawing experiments.

**Figure 3:**
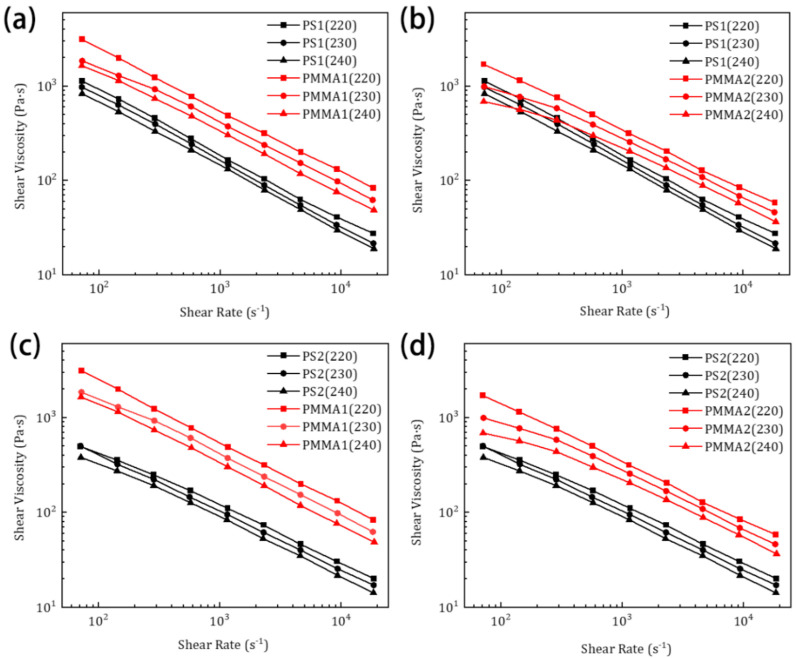
Relationships between shear viscosity and shear rate for raw materials: (**a**) PS1 and PMMA1, (**b**) PS1 and PMMA2, (**c**) PS2 and PMMA1, (**d**) PS2 and PMMA2 at 220, 230, and 240 °C.

Further, the rheological properties of all the selected materials are also tested at different shear rates and temperatures, and the relationships between the shear viscosity and shear rate are shown in Figure 3. For all the PS and PMMA materials, the shear viscosity decreases with the increase in the shear rate, showing a typical “shear thinning” effect, which is beneficial to fibre drawing. It can be found that the shear viscosity of PS1 and PMMA2 is nearest in the whole shear rate range. The shear viscosity of PMMA2 is more sensitive to the change of temperature. With the increase in temperature, the viscosity of PMMA2 decreases more obviously. In the low shear rate region, the viscosity of PMMA2 is gradually close to PS1, and the intersections of the viscosity curves of PS1 and PMMA2 at 230 and 240 °C are observed. In other words, from the point of view of rheological properties, the PS1 and PMMA2 are also suitable for co-extrusion processing under appropriate conditions. 

In addition, for the drawing process of polymer, the melt strength should be taken into account. Low melt strength is not conducive to fiber drawing and easy to break and degrade. Of course, high melt strength is also not suitable for drawing. It is probably due to that high melt strength contributes to the occurrence of unstable flow, always resulting in the formation of serrated or melts fracture. It is therefore necessary to choose the appropriate melt strength of material. Generally, the melt strength is mainly determined by the molecular structure and molecular weight of the material. As for a specific material, its melt strength can be obviously affected by the temperature. As the temperature increases, the melt strength decreases. It is found that the fiber is prone to break in the process of drawing at 240 °C, which is not conducive to continuous drawing. Instead, at 230 °C, the whole drawing process is steady, and continuity and regularity of micro/nano structure interface in the fiber present an excellent performance. Accordingly, the temperature of 230 °C is used as the operating condition during drawing process.

The experimental values of the fiber diameter obtained from the optical microscope and SEM images are summarized in Table 1. For the PMMA (shell)/PS (core) material system, the initial outer diameter of PMMA shell used for the preformed rod preparation is approximately 29 mm, while its inner diameter is 8.6 mm. During the drawing process, the outer and inner diameter of preformed rod decrease. In detail, after the first drawing step, the diameter of PS cores decreases to 180 µm with a size reduction ratio of about 48. After the second drawing step, the diameter of the PS cores obtains a value of 12 µm with a size reduction ratio of about 15. The third step reduces the PS cores diameters to about 0.8 µm with a size reduction ratio of 15. As for the PS (shell)/PMMA (core) material system, the diameter of the resulting PMMA cores after multi-drawing step exhibits various, and the corresponding values are listed in Table 1.

The factors that influence the fibre size have been previously discussed (see Ref. [5,6]). The geometric sizes of the array obtained via this method can be precisely controlled by adjusting the preform core diameter, fiber outer diameter, and the number of iterative drawing steps. Generally, the number of stacked fibres and the tightness among them also show a great impact on the ultimate size of the array structure. Using this method, porous array templates and indefinite wires with different geometric features can be designed to meet various application requirements.

**Figure 4:**
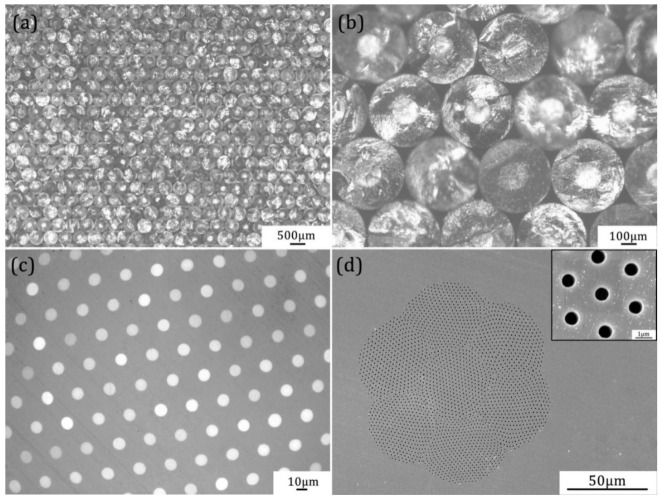
Cross-sectional images of PMMA/PS fibers of each drawing step: (**a**) bundling and stacking of 1st-draw PMMA/PS fibers, (**b**) enlarged image of 1st-draw PMMA/PS fibers, (**c**) 2nd-draw fibers, (**d**) SEM image of 3rd-draw fibers after the PS is removed, the inset is enlarged image of pores.

The cross-sectional images of the fabricated PMMA/PS fibres of each drawing step are shown in Figure 4. Figure 4a,b show the cross-sectional images with different scales of the PMMA/PS fibres which are closely stacked after the first drawing. It can be seen that the diameter distribution of the 1st-draw PMMA/PS fibres exhibits uniform, and the self-arrangement presents a regular hexagon after close stacking. The cross-sectional structure of the 2nd-draw fibres is shown in Figure 4c. It shows a high-precision hexagonal packing of the bright PS cores inside the dark PMMA matrix. Both the diameter and spacing of the PS cores are uniform. A total of seven 2nd-draw fibers are stacked into hexagonal array to conduct the third step with thermal drawing. Figure 4d shows the SEM photo of the cross-section of the 3rd-draw fibers after the PS is removed by dissolution. The SEM results indicate that the diameter of PS cores is reduced to less than 1 μm after the third thermal drawing, and further the interface between the PS cores and PMMA matrix is clear. It is also found that complete morphology and regular array structure are still maintained. Obviously, more 2nd-draw fibers or 3rd-draw fibers can be stacked for next drawing step, resulting in a significant increase in array points. Moreover, to meet the real applications, other special patterned array structures can be also designed via the same method.

**Figure 5:**
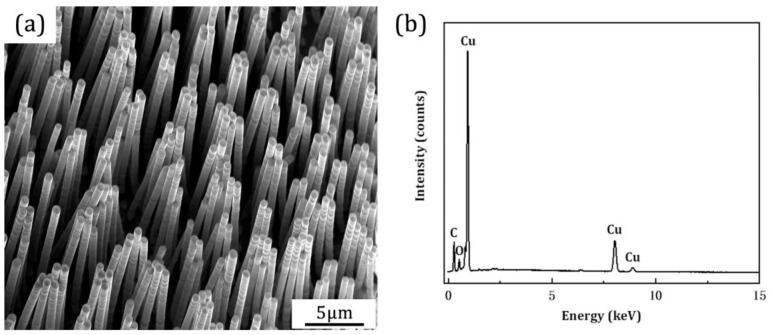
(**a**) Scanning electron microscopic images and (**b**) energy dispersive X-ray spectrum of the fabricated Cu nanowires.

In the experiment, the PMMA porous array template can be used to fabricate Cu nanowires through the processes of electrode plating, electrochemical deposition, and template removal. As shown in Figure 5a, the Cu nanowires have a columnar structure (about 800 nm in diameter and more than 10 μm in length). The diameter and length of the resulting Cu nanowires are consistent with the structure size of the PMMA template prepared by the three-step drawing. The agglomeration at the top of the nanowires can be observed due to the high aspect ratio and agglomeration effect of nanowires. The EDX spectrum detected on the nanowires also confirms that the nanowires are mainly composed of copper (Figure 5b).

**Figure 6:**
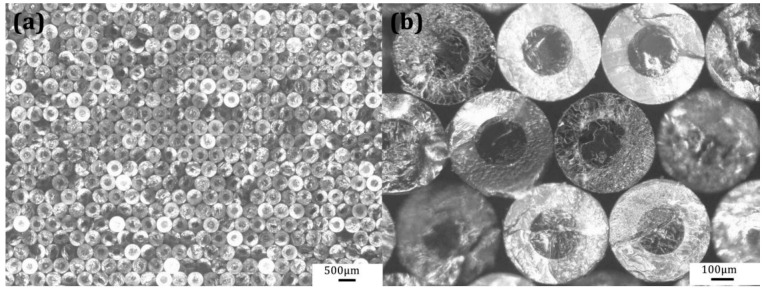
Cross-sectional images of the 1st-draw PS/PMMA fibers: (**a**) bundling and stacking of PS/PMMA fibers, (**b**) enlarged image of PS/PMMA fibers.

During the preparation of PMMA ultra-long micro/nanowires using PS (shell)/PMMA (core) material system, the morphology of the fibres after the first drawing step is depicted in Figure 6. Figure 6a is an optical microscope image of the cross-sectional of the 1st-draw PS/PMMA fibers after close stacking. A corresponding local enlarged view is shown in Figure 6b. It can be seen that, the obtained 1st-draw PS/PMMA fibers had uniform diameter distribution and regular arrangement. 

**Figure 7:**
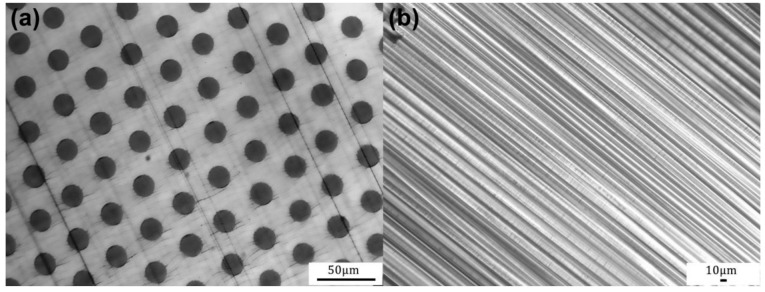
(**a**) Cross-sectional image of the 2nd-draw PS/PMMA fiber, (**b**) PMMA microwires with the diameter of about 19 μm after dissolving outer PS of the 2nd-draw PS/PMMA fiber.

The cross-sectional morphology of PS/PMMA fibers obtained by a two-step thermal drawing reduction is shown in Figure 7a. The results indicate that the dark PMMA cores array features a regular hexagonal distribution with uniform diameter and spacing in the bright PS matrix. By dissolving the 2nd-draw PS/PMMA fibers to remove the outer shell of PS, columnar long PMMA microwires with uniform diameter can be obtained, as depicted in Figure 7b. 

**Figure 8:**
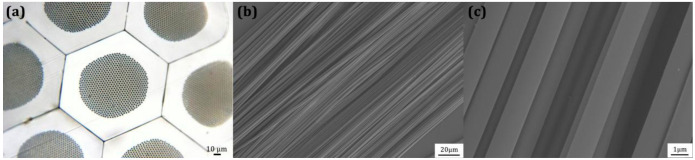
(**a**) Cross-sectional image of the 3rd-draw PS/PMMA fiber, (**b**) SEM image of PMMA microwires with the diameter of about 1.6 μm after dissolving outer PS of the 3rd-draw PS/PMMA fiber, (**c**) enlarged SEM image of PMMA microwires.

In Figure 8a, we show a perfect ordered arrays of arrays of PMMA microwires (~4000 wires of 1.6 μm) in the cross-section of the PS/PMMA composite fiber obtained after three-step thermal drawing and diameter reduction. The ultra-long PMMA microwires can be extracted from the PS matrix retaining their global alignment (Figure 8b). Remarkably, the microstructure is undistorted after the drawing steps and the surface of PMMA microwires (Figure 8c) is smooth with complete orientation structure. Moreover, the geometric size distribution of the obtained PMMA microwires is also uniform.

**Figure 9:**
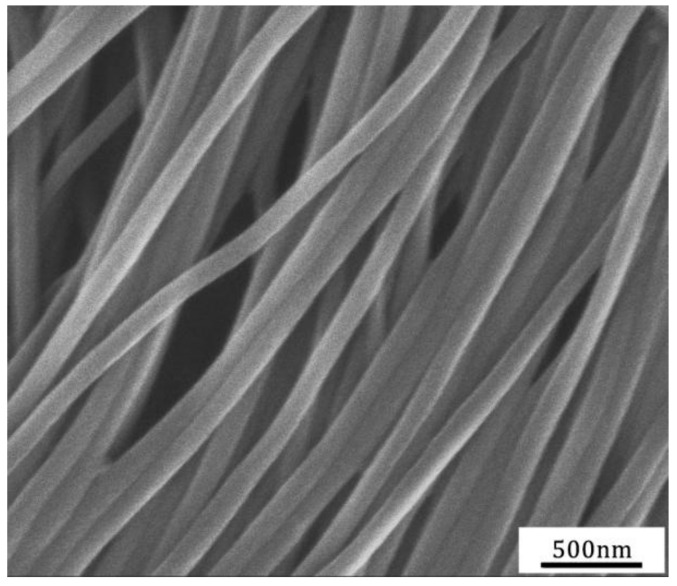
SEM image of PMMA nanowires with diameter of about 100 nm after dissolving the outer PS of 4th-draw PS/PMMA fiber.

Generally, to further reduce the size of the wires, an additional drawing step is always required. After the 4th-draw of PS/PMMA fibers, the long PMMA nanowires with a diameter of approximately 100 nm are depicted in Figure 9. Although the control over the nanoscale structure appears difficult, the wires can be regularly scaled down, and their structural integrity is also maintained. For thermoplastic polymer material systems, the limitation of ultimate size reduction mainly depends on the drawing dynamics and viscoelastic properties of the materials. Yaman et al. demonstrated that amorphous glasses and polymers that soften during drawing can be easily reduced to a radial size of 10 nm without axial breakdown. In addition, they inferred that with careful tuning and control of the feed-in and draw speeds, the drawing process can yield molecular wires. However, the ultimate size reduction limit is still an enticing question [32]. Melt co-drawing of aligned ultra-long polymer, low melting point metal, or alloy nanowires is an interesting alternative to existing chemical or other methods for producing nanowires.

## 4. Conclusions

The polymer composite fibers embedding ultra-long micro/nanowires are fabricated in this study using iterative melt co-drawing and bundling technique. PMMA porous array templates can be obtained with section-cutting of the PMMA/PS (shell/core) fiber and dissolution of inner PS. The optical microscopy and SEM pictures show that the PS cores or pores exhibit a regular hexagonal array distribution inside the PMMA matrix, indicating a uniform diameter and spacing. Based on the PMMA porous array template, the Cu nanowires are successfully prepared via electrochemical deposition. Instead, when the PS and PMMA are selected as the shell and core materials, a similar preparation process can be used to obtain PMMA ultra-long micro/nanowires. It is also found that, the surface of the PMMA micro/nanowires is smooth with complete orientation structure. Owing to the simple approach and its good controllability, various micro/nanowires can be designed and fabricated to meet the real application requirements.

## Figures and Tables

**Table 1:** Experimental values of the fibers diameter after each drawing step.

Material Systems (Shell/Core)		PMMA/PS	PS/PMMA
Preformed rods	*D*_shell_ (mm)	29	29
	*D*_core_ (mm)	8.6	10.6
1st-draw fibres	*D*_shell_ (mm)	0.5	0.48
	*D*_core_ (µm)	180	230
2nd-draw fibres	*D*_shell_ (mm)	1.4	1.6
	*D*_core_ (µm)	12	19
3rd-draw fibres	*D*_shell_ (mm)	1.6	1.6
	*D*_core_ (µm)	0.8	1.6

Note: *D*_shell_ and *D*_core_ represent the diameters of shell and core materials after the drawing procedures, respectively.

## Data Availability

Not applicable.
